# Berberine decreases plasma triglyceride levels and upregulates hepatic TRIB1 in LDLR wild type mice and in LDLR deficient mice

**DOI:** 10.1038/s41598-019-52253-y

**Published:** 2019-10-30

**Authors:** Amar Bahadur Singh, Jingwen Liu

**Affiliations:** Department of Veterans Affairs Palo Alto Health Care System, Palo Alto, California, 94304 USA

**Keywords:** Molecular biology, Molecular biology, Transcription, Transcription, Diseases

## Abstract

*TRIB1* is a GWAS locus associated with plasma cholesterol and triglycerides (TG) levels. In mice, liver-specific overexpression of TRIB1 lowers plasma lipid levels. Berberine (BBR) is a natural lipid lowering drug that reduces plasma LDL-cholesterol (LDL-C), total cholesterol (TC) and TG in hyperlipidemic patients and in mice by mechanisms involving upregulation of hepatic LDL receptor (LDLR). Here, we demonstrated that BBR treatment reduced plasma LDL-C, TC and TG in LDLR wildtype (WT) mice fed a high fat and high cholesterol diet and it only lowered TG in LDLR WT mice fed a normal chow diet. In hypercholesterolemic LDLR deficient mice (*Ldlr*^−/−^), BBR treatment reduced plasma TG levels by 51% compared to the vehicle control without affecting plasma cholesterol levels. Hepatic gene expression analysis revealed that *Trib1* mRNA levels were significantly elevated by BBR treatment in all three mouse models and increases of *Trib1* mRNA expression were associated with reduced expression of lipogenic genes including *Cebpa*, *Acc1* and *Scd1*. *In vitro* studies further demonstrate that BBR induces *TRIB1* mRNA expression by a transcriptional mechanism via ERK signaling pathway. These new findings warrant future *in vivo* studies to determine the causal role of Trib1 in BBR-mediated TG lowering independent of LDLR regulation.

## Introduction

Coronary artery disease (CAD) is the leading cause of morbidity and mortality in industrialized countries, and the prevalence of these diseases is rapidly increasing in developing nations^1^. Epidemiological studies have repeatedly demonstrated that elevated levels of circulating low-density lipoprotein cholesterol (LDL-C), total cholesterol (TC) and triglyceride (TG)-rich remnant lipoproteins have strong associations with the development of CAD and myocardial infarction (MI)^2–4^.

Recent genome-wide association studies (GWAS) have identified tribbles1 (*TRIB1*) functional association with risk of CAD. *TRIB1* emerged in several GWAS as a novel cardiovascular locus, where the protective allele is strongly associated with decreased levels of circulating LDL-C, TG and increased levels of high-density lipoprotein cholesterol (HDL-C) as well as with reduced incidence of CAD and MI^5,6^. In addition to this beneficial lipid profile, *TRIB1* locus has been linked to nonalcoholic fatty liver disease (NAFLD) that is characterized by the accumulation of fat in the liver^7^. A functional study using *TRIB1*-deficient mice (*Trib1*^−/−^) and hepatic TRIB1 overexpression experiment confirmed the involvement of TRIB1 in hepatic lipogenesis, which affects very low-density lipoprotein (VLDL) production through the modulation of genes involved in TG biosynthesis^8^. Another study also reported adenovirus mediated TRIB1 overexpression and knockdown resulted in reduction and increase in plasma TG, TC and subsequent changes in hepatic TG and glycogen levels^9^. However, despite the substantial evidence linking TRIB1 with dyslipidemia in humans, currently little is known about the regulation of endogenous TRIB1 expression and its relationship with nutritional factors.

Our laboratory and other investigators have previously demonstrated that berberine (BBR), a natural cholesterol-lowering compound, enhances hepatic LDL receptor (LDLR) expression through a complementary mechanism of increased stability of the transcript and suppression of HNF1α-mediated PCSK9 gene transcription^10–16^. In hyperlipidemic patients and in hyperlipidemic mice and hamsters, BBR treatments effectively lower circulating cholesterol and triglyceride levels. However, up to today, one important question that remains to be answered is whether upregulation of LDLR is prerequisite for the hypolipidemic effects of BBR. Furthermore, what are other cellular mechanisms responsible for BBR-mediated TG reduction?

Considering the beneficial effects of TRIB1 expression to plasma and hepatic lipid profile, in this study, we examined effects of BBR on liver TRIB1 expression with connections to BBR exerted hypolipidemic effects in hypercholesterolemic and normolipidemic LDLR wildtype (WT) mice and in LDLR deficient mice (*Ldlr*^−/−^). We found that BBR treatment effectively increased hepatic *Trib1* mRNA expressions in both LDLR WT and deficient mice, which were consistently associated with reduced serum TG levels in all three mouse models whereas reduction of plasma cholesterol was only observed in LDLR WT mice fed a high fat and high cholesterol diet (HFHCD). Further mechanistic studies conducted in hepatic cells demonstrated that BBR increases *TRIB1* gene transcription and promoter activity, and these effects are specifically abrogated by inhibiting ERK signaling pathway. In addition, we investigated the interrelationship between LDLR and TRIB1 expressions. We demonstrated that changes in TRIB1 expression levels do not impact on hepatic *LDLR* mRNA or LDLR protein expressions regardless of BBR treatment. Altogether we have identified *Trib1* as an important target gene induced by BBR in liver tissue and in cultured human hepatic cells. These new findings warrant future *in vivo* studies to determine the key role of Trib1 in BBR-mediated TG lowering independent of LDLR regulation.

## Results

### Reduction of serum cholesterol and triglyceride levels in hyperlipidemic mice treated with BBR

**Figure 1:**
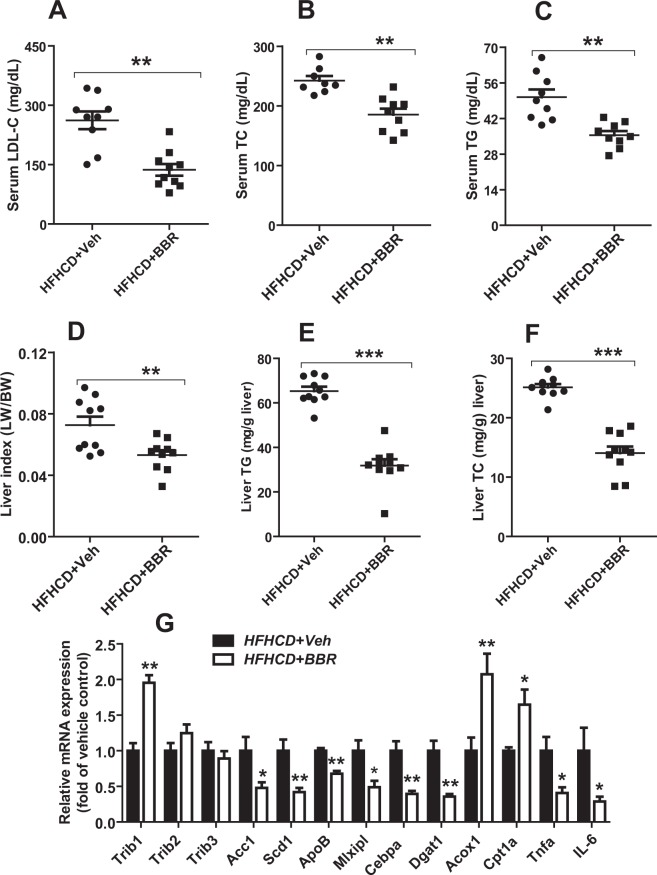
BBR increases *Trib1* mRNA levels and reduces circulating serum lipid levels in hyperlipidemic mice. Mice fed a HFHCD diet were treated with BBR (200 mg/kg, n = 10) or with vehicle (n = 10) for 14 days. At the end of drug treatment all the mice were sacrificed for serum and liver tissue collection. (**A**–**C**) LDL-C, TC and TG levels in mice sera were measured after the treatment with BBR or the vehicle. (**D**) Liver index was measured in BBR or the vehicle treated mice. (**E**–**F**) Liver TG and TC contents were measured in individual liver sample treated with BBR or the vehicle. (**G**) Quantitative real-time PCR were used to determine the relative expression levels of *Trib1* and other hepatic genes after normalization with *Gapdh* mRNA levels. Values are the mean ± S.E.M. of 10 samples per group. *P < 0.05, **P < 0.01, and ***P < 0.001, compared with the vehicle control group.

To examine LDLR dependent and independent hypolipidemic effects of BBR in mice, first, LDLR WT mice fed a HFHCD for two weeks were treated with BBR (200 mg/kg/day) (n = 10) or vehicle (n = 10) for 14 days. BBR treatment reduced serum LDL-C levels by 51% (p < 0.01), lowered serum TC levels by 28% (p < 0.01) and lowered serum TG levels by 23% (p < 0.01) as compared to the control group (Fig. 1A–C). While body weight and food intake were not affected by BBR, BBR treated hyperlipidemic mice exhibited a 27% (p < 0.01) decrease in liver index (liver weight to body weight ratio) as compared with vehicle control (Fig. 1D).

Measurement of hepatic lipids showed a significant decrease in hepatic TG (51.3%, p < 0.001) and in hepatic TC content (44.1%, p < 0.001) in BBR treated group compared to vehicle control group (Fig. 1E,F). Hepatic gene expression analysis by qPCR revealed a 1.96-fold higher *Trib1* mRNA levels in BBR-treated mice compared to the control mice. In contrast to *Trib1*, mRNA levels of *Trib2* and *Trib3* did not differ between BBR and vehicle groups (Fig. 1G). It has been reported that TRIB1 down regulates C/EBPα post transcriptionally that leads to suppression of C/EBPα target lipogenic genes such as *Scd1* and *Dgat1*^9,17^. Consistent with literature reports, we observed that in livers of BBR-treated mice, the increased *Trib1* gene expression was accompanied by attenuated expression of several lipogenic genes (*Acc1, Scd1, ApoB, Mlxipl, Cebpa, Dgat1*) and elevated expression of fatty acid β-oxidative genes *Acox1* and *Cpt1a*. BBR treatment also reduced mRNA levels of inflammatory marker genes *Tnfa* and *Il-6* whose expression was induced by the HFHCD. These results provided the first evidence demonstrating the induction of *Trib1* gene expression by BBR in liver tissue of hyperlipidemic mice expressing functional LDLR.

### Induction of hepatic *Trib1* mRNA expression and lowering serum TG by BBR in chow fed mice

**Figure 2:**
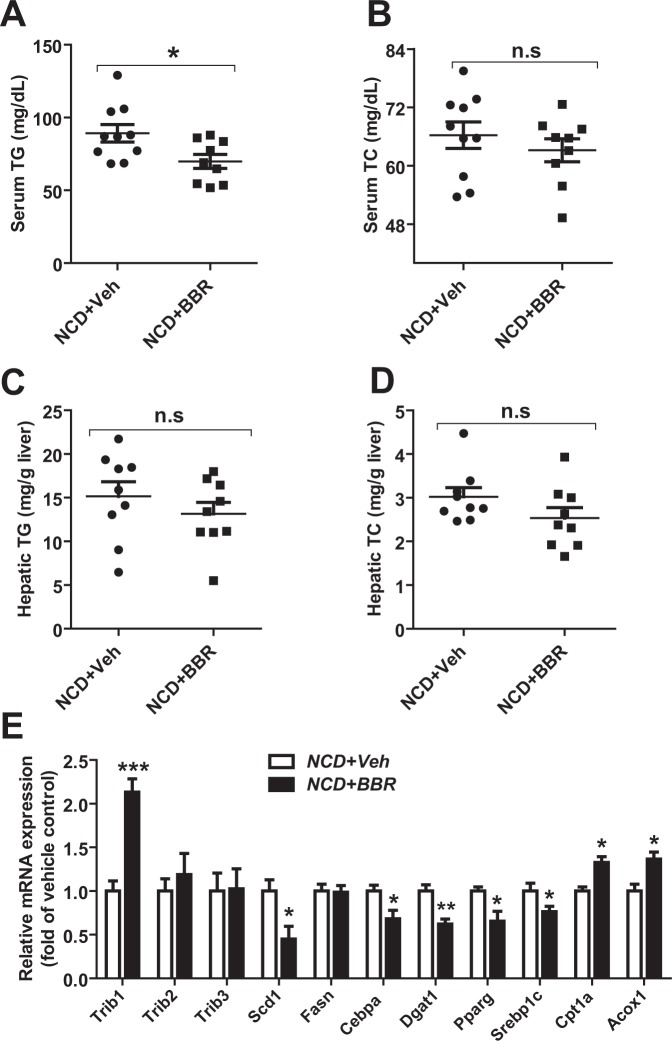
Induction of *Trib1* mRNA levels in normolipidemic mice treated with BBR. Mice fed an NCD were administered with BBR (200 mg/kg, n = 10) or the vehicle (n = 10) for 14 days. Four h fasted blood and liver samples were collected at the end of treatment. (**A,B**) Serum TG and TC levels were measured after treatment with BBR or the vehicle. (**C,D**) Hepatic TG and TC contents were measured in individual liver samples treated with BBR or the vehicle. (**E**) Quantitative real-time PCR were used to determine the relative expression levels of *Trib1* and other hepatic genes after normalization with *Gapdh* mRNA levels. Values are the mean ± S.E.M. of 10 samples per group. *P < 0.05, **P < 0.01, and ***P < 0.001, compared with the control group.

Next, we examined the hypolipidemic effect of BBR in LDLR WT mice fed a normal chow diet (NCD). Mice were treated with BBR (200 mg/kg/day) for 14 days. In these normolipidemic mice, BBR treatment lowered serum TG levels by 20% (p < 0.05) without affecting serum TC levels or hepatic lipid contents (Fig. 2A–D). Importantly, this TG lowering effect was accompanied by a 2.1-fold increase in *Trib1* mRNA level in the liver (Fig. 2E). Despite the unchanged hepatic lipid levels, we observed consistent changes in reducing lipogenic genes and elevating FA β-oxidative genes by BBR treatment (Fig. 2E) to similar extents of that in BBR treated HFHCD fed mice.

### Induction of hepatic *Trib1* mRNA expression by BBR is independent of LDLR status and is associated with TG lowering

**Figure 3:**
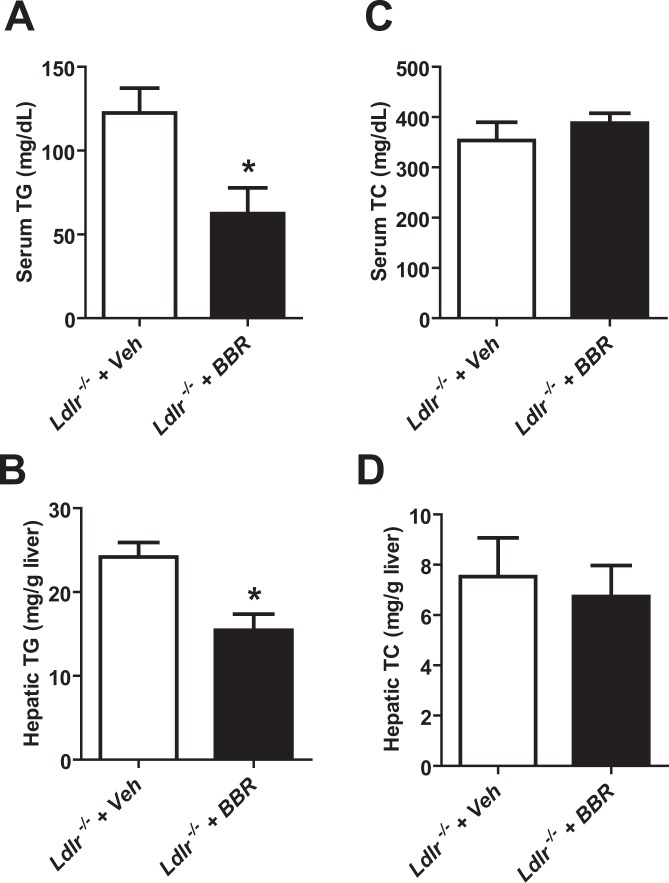
BBR reduces serum TG levels and liver TG contents in hypercholesterolemic *Ldlr* ^−/−^ mice. NCD-fed *Ldlr* ^−/−^ mice were treated with BBR (150 mg/kg, n = 3) or the vehicle (n = 3) for 7 days. At the end of treatment, the mice were fasted 4 h for blood and liver tissue collection. (**A,C**) Serum TG and TC levels were measured after 7 days of BBR or the vehicle treatment. (**B,D**) Hepatic TG and TC contents were estimated in individual liver samples of BBR or the vehicle treated *Ldlr*^−/−^ mice. Data shown are mean ± S.E.M. of 3 liver samples per group. Significance was determined between vehicle and BBR treated group by Student’s t test (*P < 0.05).

To further examine the relationship between serum TG reduction and *Trib1* upregulation, we utilized LDLR-deficient mice (*Ldlr*^−/−^). In this hypercholesterolemic mouse model, BBR treatment for 1 week at a daily dose of 150 mg/kg, led to a significant reduction in serum TG levels (−51.3%, p < 0.05) (Fig. 3A) and hepatic TG content (−36.3%, p < 0.05) (Fig. 3B). As we predicted, serum cholesterol levels were unchanged by BBR treatment, that validated the LDLR-dependent effect of BBR in reducing serum LDL-C. Consistent with unchanged serum TC, hepatic cholesterol content was not affected by BBR treatment in these LDLR deficient mice.

**Figure 4:**
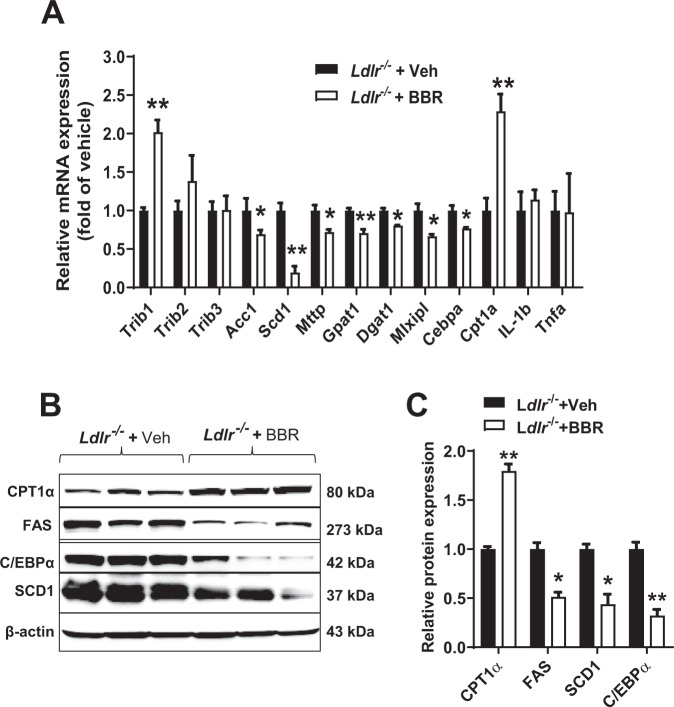
Induction of *Trib1* mRNA levels in hypercholesterolemic *Ldlr* ^−/−^ mice treated with BBR. (**A**) Quantitative real-time PCR were used to determine the relative expression levels of *Trib1* and other hepatic genes after normalization with *Gapdh* mRNA levels. (**B,C**) Total protein lysate were prepared from individual liver samples of each group. Equal amounts of homogenate proteins (70 μg) were resolved by SDS-PAGE and CPT1α, FAS, C/EBPα, and SCD1 protein were detected by immunoblotting. Membranes were reprobed with anti-β-actin antibody. Indicated protein abundances were quantified and normalized by signals of β-actin. The data shown are mean ± S.E.M. of three liver samples per group. Significance was determined in all panels by Student’s t test (*P < 0.05, **P < 0.01*)*.

Hepatic gene expression analysis showed a significant 2-fold increase in *Trib1* mRNA levels (p < 0.001) with BBR treatment in *Ldlr*^−/−^ mice, again no changes in mRNA levels of *Trib2* and *Trib3* were observed (Fig. 4A). Hepatic *Trib1* upregulation subsequently led to the reduced mRNA expression of key lipogenic genes (*Acc1, Scd1, Mttp, Gpat1, Dgat1, Mlxipl, and Cebpa*) (Fig. 4A**)**. We further examined the protein expression of TRIB1 regulated lipogenic genes and observed substantial decreases in hepatic protein levels of C/EBPα, SCD1 and FAS and the increased expression of CPT1α protein in the liver of BBR-treated mice.

Taken together, these study results of three mouse models suggest that hypolipidemic effects of BBR are mediated through LDLR-dependent and independent mechanisms. Induction of *Trib1* expression in the liver is strongly associated with BBR-mediated plasma TG reduction.

### BBR induces *TRIB1* mRNA expression in HepG2 Cells and in human primary hepatocytes (HPHs)

**Figure 5:**
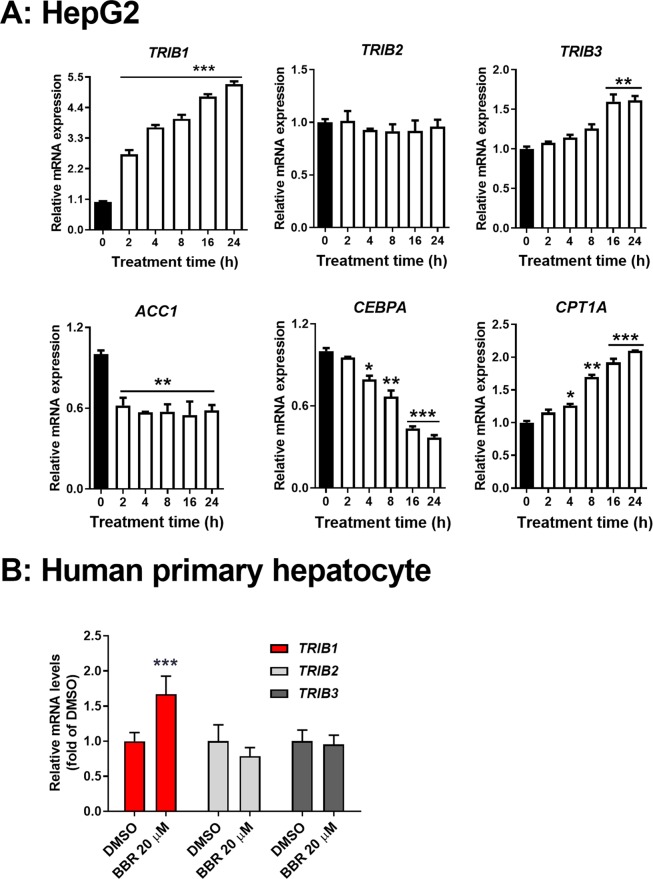
Induction of *TRIB1* gene expression and downregulation of lipogenic genes by BBR in human liver cells. (**A)** HepG2 cells were seeded in 12-well cell culture plate (5 × 10^5^cell/well) and treated with BBR (40 μM) in duplicate wells for different time intervals as indicated. Total RNA was isolated from treated cells for real-time PCR analysis. The data shown are mean ± S.E.M. of two RNA samples with triplicate measurement per RNA sample. **(B)** HPHs were treated with BBR (20 μM) or DMSO for 3 h in triplicate wells. *TRIB1* mRNA levels were determined by real-time PCR. The data shown are mean ± S.E.M. of three RNA samples with triplicate measurements per RNA sample. *P < 0.05, **P < 0.01, and ***P < 0.001, compared with DMSO control.

The upregulation of LDLR by BBR via mRNA stabilization has been thoroughly studied in human hepatoma derived HepG2 cells in our previous studies^10,11^. Thus, to further characterize the molecular mechanism regulating *TRIB1* gene expression, we treated HepG2 cells with BBR for different time intervals and we found that *TRIB1* mRNA levels were rapidly elevated upon BBR treatment and a 2.7-fold increase was detected at 2 h and it reached to a maximal level of 5.3-fold over baseline by 24 h (Fig. 5A). *TRIB3* mRNA levels were slightly increased at later time points of BBR treatment while *TRIB2* mRNA levels remained unchanged during the study course. Importantly, BBR treatment down regulated *CEBPA* and its target genes in a time course largely consistent with *TRIB1* upregulation. The rapid elevation of *TRIB1* mRNA levels upon BBR treatment was further confirmed in HPHs treated with BBR for 3 h (Fig. 5B).

**Figure 6:**
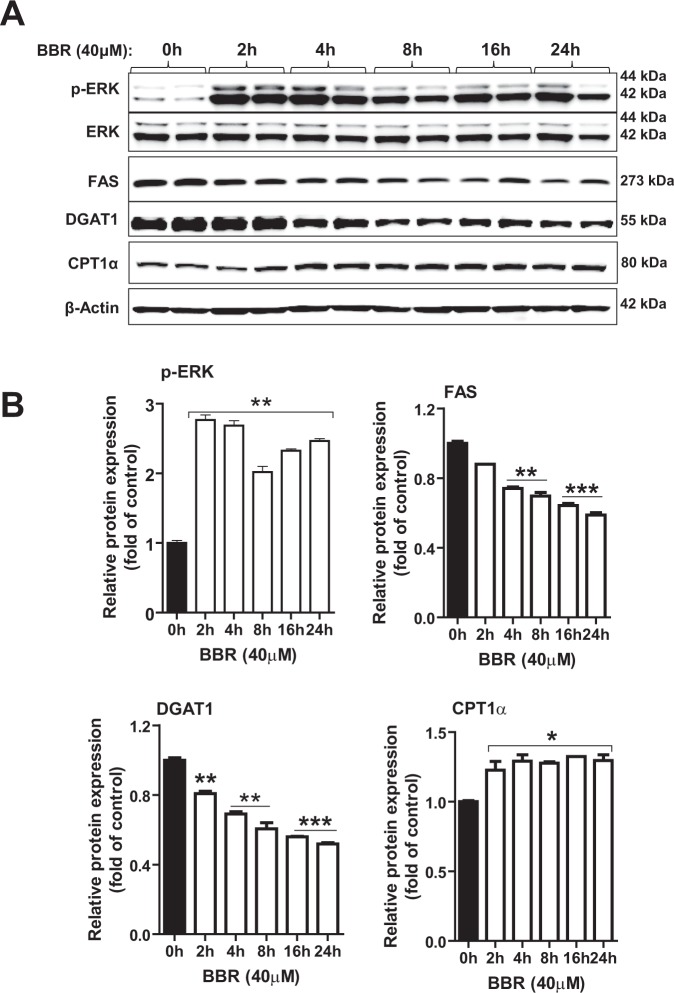
Time-dependent induction of ERK phosphorylation and suppression of lipogenic gene products by BBR in HepG2 cells. HepG2 cell were treated with 40 μM BBR or vehicle DMSO for the indicated times in duplicate wells. **(A)** Total cell lysates were isolated for Western blot analysis of indicated proteins. (**B**) Protein levels of interest were normalized to levels of β-actin.

It was recently reported that some novel compounds termed as TRIB1 inducers were discovered through a compound library screening, these TRIB1 inducers upregulate *TRIB1* mRNA levels in HepG2 cells by an ERK dependent mechanism^18,19^. To determine the involvement of ERK signaling pathway in BBR-mediated *TRIB1* induction, first, we treated HepG2 cells with BBR in different time points and conducted Western blot analysis to detect activated and phosphorylated ERK (p-ERK). As shown in Fig. 6, the signal intensity of p-ERK was 2.8- fold higher than the baseline after 2 h BBR treatment and p-ERK signal intensity remained elevated throughout the time course of 24 h. Since we could not directly detect endogenous TRIB1 protein expression by Western blotting owing to the extremely low expression level^20^, we analyzed the protein abundance of TRIB1 modulated genes FAS and DGAT1. Like the qPCR results, BBR treatment reduced these protein levels as early as 2 h and reached lowest expression after 24 h treatment. In addition, we detected elevated protein abundance of CPT1α in BBR treated cells, which confirmed the qPCR results (Fig. 5A). Altogether, these results from cultured hepatic cells validated our findings obtained from liver tissues and further demonstrate that BBR activates ERK signaling pathway and upregulates hepatic *TRIB1* gene expression, resulting in the down regulation of TRIB1 modulated lipogenic genes.

### BBR induces *TRIB1* mRNA expression by a transcriptional mechanism via ERK signaling cascade

**Figure 7:**
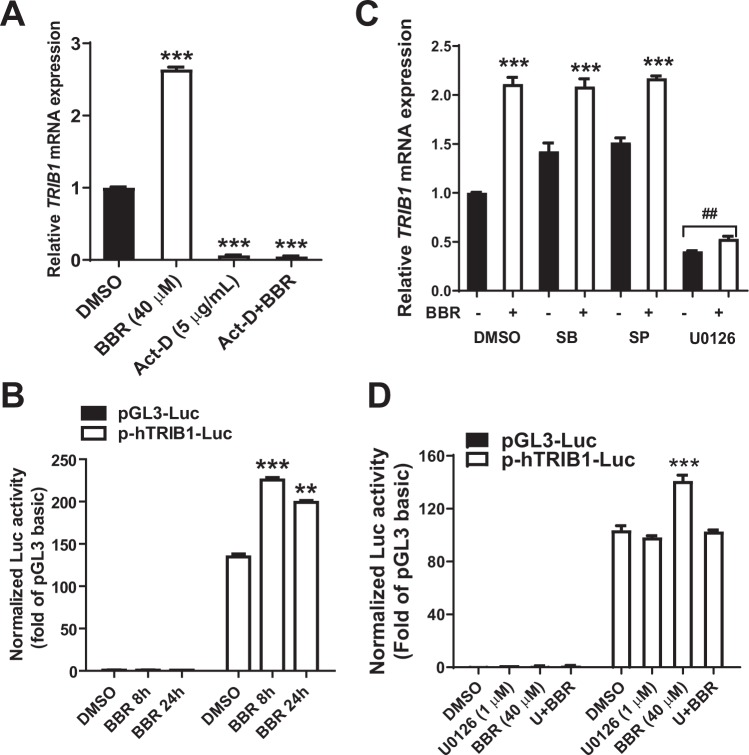
BBR activates *TRIB1* gene transcription and promoter activity in an ERK-dependent manner. (**A)** HepG2 cells were pre-treated with Act-D (5 μg/ml) for 30 min and subsequently treated with BBR or DMSO for 5 h. Total RNA was isolated for gene expression analysis by real-time PCR. After normalization with *GAPDH* mRNA levels, the relative expression of *TRIB1* mRNA in DMSO treated cells was set at 1. ***P < 0.001, compared with DMSO control. **(B**) HepG2 cell were seeded in 96 well plate and transfected with *TRIB1* promoter luciferase reporter (p-hTRIB1-Luc) or pGL3 control vector, and cotransfected with pCMV-β-gal and treated with DMSO or BBR for 8 h and 24 h prior to cell lysis for luciferase and β-gal assays. Triplicate wells were used for each condition. **P < 0.01 and ***P < 0.001, compared with DMSO control. **(C)** HepG2 cells were pre-treated with the indicated MAPK inhibitors (SB, 10 μM SB203580; SP, 10 μM SP600125; U0126, 1 μM) for 30 min prior to the BBR treatment of 8 h at 40 μM concentration. RNA was harvested and subjected to real-time PCR to assess *TRIB1* levels and normalized with *GAPDH* mRNA levels. Asterisks indicate statistically significant inductions of samples treated with inhibitors with or without BBR as compared to DMSO control. ^##^ indicated significant reductions of *TRIB1* mRNA levels compared to DMSO control (p < 0.01). (**D**) *TRIB1* promoter luciferase activities were assayed in DMSO or BBR-treated cells in the presence of absence of U0126. The data shown are representative of three separate transfection experiments with similar results. ***P < 0.001, compared with DMSO control.

Next, we determined whether the induction of *TRIB1* by BBR is through increased gene transcription by utilizing Actinomycin-D (Act-D), a general inhibitor of transcription. HepG2 cells were treated with BBR for 5 h in the absence or the presence of Act-D. Act-D treatment greatly attenuated *TRIB1* gene transcription and totally abolished the inducing effect of BBR (Fig. 7A). To further demonstrate a transcriptional regulatory mechanism, we cloned human *TRIB1* proximal promoter region from −840 to + 127 relative to the 5’end of exon 1 into luciferase reporter pGL3-basic, generating *TRIB1* promoter reporter p-hTRIB1-Luc. *TRIB1* reporter plasmid and control reporter pGL3-basic along with pCMV-β-Gal (a transfection normalizing plasmid) were transfected into HepG2 cells for 24 h and subsequently transfected cells were treated with BBR for 8 h and 24 h. The normalized luciferase activity in p-hTRIB1-Luc transfected cells was nearly 130-fold of that of control vector pGL3-basic, indicating that this is a highly active promoter. BBR treatment did not change the luciferase activity of pGL3-basic but significantly increased the *TRIB1* promoter activities at both time points (Fig. 7B). These results, combined with the Act-D experiment, clearly demonstrate that BBR positively regulates *TRIB1* gene transcription.

To further identify the signaling pathways contributing to *TRIB1* induction, we utilized inhibitors targeting individual MAPK pathways (p38 MAPK inhibitor SB203580, JNK inhibitor SP600125 and MEK1 inhibitor U0126). HepG2 cells were pretreated with different MAPK inhibitors for 30 min and subsequently treated with BBR for 5 h. Among three inhibitors applied, only the ERK1/2 upstream kinase MEK1 inhibitor U0126 was able to abrogate BBR-mediated induction of *TRIB1* (Fig. 7C). Blocking ERK signaling pathway not only abolished the inducing effect of BBR but also lowered the basal level of *TRIB1* mRNA expression, suggesting a tight regulation of *TRIB1* gene expression by the ERK signaling pathway. SB203580 and SP600125 treatment modestly increased *TRIB1* mRNA basal levels, suggesting that the p38 MAPK and JNK pathways may function as negative regulators of *TRIB1* transcription and/or mRNA stability, however, blocking p38 and JNK signaling pathways did not attenuate BBR-mediated transcriptional activation of *TRIB1* (Fig. 7C). We further showed that the inducing effect of BBR on *TRIB1* promoter activity was lost in the presence of U0126 (Fig. 7D). Taken together, these *in vitro* studies demonstrate a transcriptional effect of BBR on *TRIB1* gene, and this effect is a downstream event of BBR-elicited activation of the ERK signaling cascade.

### Hepatic TRIB1 overexpression or knockdown does not impact LDLR expression levels

As aforementioned, we observed the upregulation of hepatic *Trib1* mRNA expression by BBR in LDLR WT and in LDLR deficient mice, demonstrating an LDLR independent regulatory effect. Next, we examined the impact of altered TRIB1 expression on LDLR abundance in liver cells. First, we transfected mouse primary hepatocytes (MPHs) with an adenovirus expressing human TRIB1 (Ad-hTRIB1) or the control adenovirus expressing GFP at various MOIs. As shown in Supplemental Fig. 1A, qPCR measurements demonstrated marked increases in *TRIB1* mRNA levels in a MOI dose-dependent manner but mRNA levels of *Ldlr* as well as *Pcsk9* remained unchanged. Western blotting results (Supplemental Fig. 1B) further corroborated the gene expression analysis. Although the anti-TRIB1 antibody failed to detect endogenous TRIB1, it recognized overly expressed TRIB1 in a MOI dose-dependent manner.

In the second set of experiments, we transduced HepG2 cells with Ad-hTRIB1 and Ad-sh-hTRIB1 that expressed a shRNA targeting human *TRIB1*. The adenovirus (Ad-sh-mTRIB1) expresses a shRNA which targets only mouse *Trib1*, and we included that as a negative control in addition to the control adenovirus (Ad-GFP). Supplemental Fig. 1C shows that changing TRIB1 expression levels in HepG2 cells by overexpression or knockdown did not affect LDLR and PCSK9 protein levels in total cell lysates. We also detected secreted PCSK9 in the medium that was unaffected by TRIB1 expression status. Supplemental Fig. 1D shows quantitative results of LDLR and PCSK9 protein expressions. Gene expression analysis by qPCR confirmed the increased mRNA expression of *TRIB1* mRNA in Ad-hTRIB1 transduced cells and the diminished *TRIB1* mRNA levels in Ad-sh-hTRIB1 transduced cells to 60% of the control (Ad-GFP). Again, *LDLR* mRNA remained unchanged (Supplemental Fig. 2). Taken together, these *in vitro* results revealed a disconnection between TRIB1 and LDLR, which is in line with our *in vivo* results demonstrating that BBR exerts TG lowering effects regardless of LDLR expression status.

**Figure 8:**
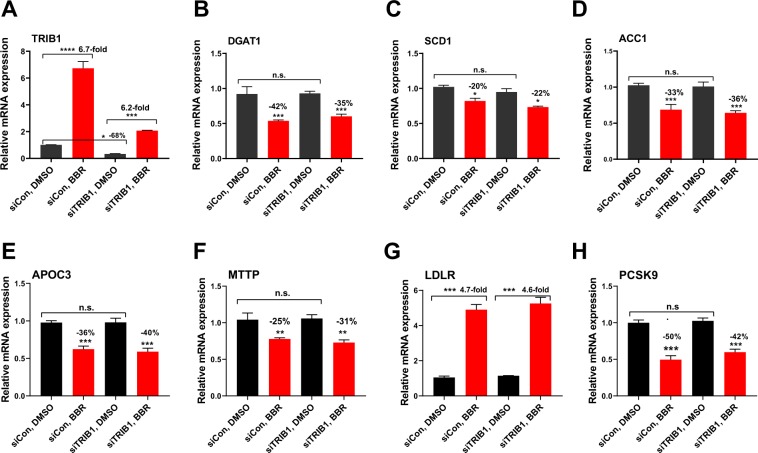
Depletion of TRIB1 by siRNA transfection had no effects on basal and BBR-mediated suppression of lipogenic genes. HepG2 cells in triplicated wells were transfected with control siRNA (siCON) or siTRIB1 for 48 h and BBR (40 μM) or DMSO were added to the transfected cells for the last 12 h before RNA isolation and subsequent qPCR analysis of indicated genes. Statistical significance was determined with One-way ANOVA with Tukey’s multiple comparisons test. **p < *0.01* and ***p < *0.001* compared with the control siRNA transfected cells treated with DMSO.

In addition to examine the effects of *TRIB1* knockdown on LDLR by Ad-shTRIB1 transduction, utilizing siRNA mediated gene silencing, we attempted to examine the effects of *TRIB1* knockdown on BBR-mediated suppression of lipogenic genes. We transfected HepG2 cells in triplicate wells with either scrambled siRNA (siCon) control or with a pool of four siRNAs targeted to human *TRIB1* (siTRIB1) for 48 h with the absence or presence of BBR in the last 12 h before cell lysis. Compared to control siRNA, transfection of siTRIB1 pool significantly reduced *TRIB1* mRNA levels nearly 70%. (Fig. 8A). However, the mRNA levels of several lipogenic genes including *DGAT1*, *SCD1*, *ACC1, APOC3 and CEBPA* were not significantly changed in siTRIB1 transfected cells (Fig. 8B–F). Furthermore, BBR induced suppression of these lipogenic genes were also not affected by *TRIB1* knockdown. Regarding LDLR and PCSK9 mRNA expression, the siRNA-mediated depletion of TRIB1 recaptured the results of adenovirus-mediated knockdown without affecting basal and BBR-regulated expressions (Fig. 8G,H). These data were largely similar to the results of a previously published report which showed that the repressive activity of TRIB1 inducer BRD0418 on lipogenic gene expression in HepG2 cells were not attenuated by *TRIB1* genetic deletion^18^.

## Discussion

Since the discovery of *TRIB1* locus as a novel locus having a strong association with human plasma and hepatic lipid metabolism by several GWAS reports^5–7^, TRIB1 has been considered as a therapeutic target for developing new approaches to treat cardiometabolic diseases^19,20^. In this study, we report that the hepatic transcription of *Trib1* is induced by the natural cholesterol lowering compound BBR in three different mouse models and *Trib1* induction correlated strongly with plasma TG reduction regardless of the LDLR expression status.

It has been well documented by several clinical studies that BBR treatment reduces plasma LDL-C, TC and TG in hyperlipidemic patients^21^. Mechanistic studies conducted in our laboratory and by other investigators revealed that BBR has a unique ability to upregulate hepatic LDLR through two complementary mechanisms of prolonging *LDLR* transcript^10,11^ and simultaneously inhibiting the transcription of *PCSK9*^14–16^, the degrader of LDLR protein. This regulatory mechanism on LDLR explains the LDL-C reduction in patients and in animal models. In addition to lowering TC and LDL-C, BBR lowers plasma TG significantly and reduces hepatic steatosis^22^. Currently, the molecular mechanisms underlying the BBR-mediated TG reducing effects are not fully understood.

To gain a better understanding of the BBR-mediated hypolipidemic effects, in this study we tried to identify potential cellular regulators that participate in BBR-induced changes in plasma lipid metabolism independent of LDLR. To achieve this goal, we utilized three different mouse models including hyperlipidemic LDLR WT mice, normolipidemic LDLR WT mice and the NCD-fed LDLR deficient mice (*Ldlr*^−/−^) that are naturally in a hypercholesterolemic state.

Our study results showed that LDL-C and TC lowering effects of BBR were prominent in the LDLR WT mice under a hyperlipidemic condition but not in the NCD-fed mice, presumably owing to the low LDL-C levels in these mice. Importantly, we demonstrated that in the hypercholesterolemic LDLR deficient mice in which serum TC reached a level of over 350 mg/dL, higher than the WT mice fed a cholesterol enriched diet, BBR treatment did not affect the serum cholesterol levels at all, but BBR effectively lowered plasma TG by 51% of the control and lowered hepatic TG content by 36% in *Ldlr*^−/−^ mice. Thus, our study results provided direct evidence supporting the mechanism of action for BBR-mediated reduction of serum cholesterol through upregulating the liver LDLR.

Comparing results from three mouse studies, it became clear that BBR treatment resulted in significant serum TG reductions that are not entirely associated with serum TC levels nor with liver LDLR expression status. Gene expression analysis from all liver samples of the three mouse studies revealed a consistent and approximately a 2-fold elevation of *Trib1* mRNA levels that were accompanied by reduced expression of several TRIB1 modulated lipogenic genes.

To further establish the role of TRIB1 in the beneficial effects of BBR on plasma and hepatic lipid metabolism, we utilized HPHs and HepG2 cells. In the cultured liver cells, BBR induced a rapid increase in *TRIB1* mRNA levels. Different from the regulatory mechanism of BBR on LDLR mRNA stabilization, BBR increases *TRIB1* mRNA expression at the transcriptional level evident by the loss of its effect in the presence of Actinomycin D. By generating a human *TRIB1* promoter reporter, we further located the BBR-inducing effect to the proximal 1 kb region upstream from the exon 1. Further deletion analysis to map the cis-regulatory elements within this promoter region is undertaken in our laboratory to identify transcription factors that are involved in the BBR-induced *TRIB1* gene transcription.

Based upon cumulative literature reports, TRIB1 has two well-defined functions of promoting C/EBPα protein degradation via ubiquitination and facilitating MEK1-mediated phosphorylation of ERK^23^. Interestingly, TRIB1 not only enhances ERK phosphorylation, *TRIB1* gene transcription is regulated by ERK1^24^, possibly through a feedforward mechanism. Our studies in liver cells also confirm that inhibition of ERK signaling pathway by MEK1 specific inhibitor U0126 greatly reduces *TRIB1* mRNA levels and abrogates the inducing effect of BBR. Our findings using BBR as a tool are consistent with other small molecules that induce *TRIB1* mRNA expression^19,20^. It is worth noting that BBR was shown to induce *TRIB1* mRNA expression alongside the *TRIB1* inducers in HepG2 cells^19^. Our study and the previous report on *TRIB1* inducers both point to the importance of ERK signaling pathway in regulating *TRIB1* gene transcription.

Our *in vivo* studies have clearly demonstrated that BBR induction of *Trib1* in the liver is not dependent on LDLR expression status. Regarding the relationship of TRIB1 and LDLR expression, it has been reported that in mice, adenoviral mediated over expression of TRIB1 increased liver LDLR expression due to reduced plasma PCSK9 levels^25^. We tried to replicate that study in cultured liver cells. Our results showed that in both MPH and HepG2 cells, adenoviral mediated TRIB1 overexpression or knockdown had no impact on *LDLR* mRNA or LDLR protein levels, and we did not observe significant changes in PCSK9 intracellular and extracellular levels under those conditions. The discrepancy between our results and the previous mouse study could reflect the different assay system of *in vivo* and *in vitro*.

To further understand the relationship between TRIB1 induction and suppression of lipogenic genes upon BBR treatment, we used *in vitro* system to knockdown *TRIB1* in HepG2 cells with highly specific *TRIB1* siRNAs. QRT-PCR analysis demonstrated the efficacy of siTRIB1 with a 68% reduction of *TRIB1* transcript, yet we did not detect significant changes in *DGAT1, SCD1, ACC1*, *APOC3* and *CEBPA* mRNA basal levels. This was unexpected as a previous report has shown that transfection of si-TRIB1 in Huh7 cells effectively reduced c/EBPa protein levels^20^. Moreover, we did not detect changes in BBR mediated suppression of these lipogenic genes. This disconnection could be explained by several factors. First, given the established effects of TRIB1 overexpression on lipogenic genes, it is possible that BBR-mediated induction of *TRIB1* contributes to the suppression of lipogenic genes to some extents, but other mechanisms such as activation of AMP kinase by BBR^26^ could also account for the reduction of lipogenesis in BBR treated cells. Secondly, our experiments of siTRIB1 transfection and the Ad-shTRIB1 transduction in HepG2 cells only achieved partial depletions of TRIB1 which might be responsible for the lack of abolishing effect to BBR. Thirdly, under TRIB1 deficient condition, BBR-stimulated other signaling pathway such as AMPK activation could evoke a compensatory response. A compensatory response in HepG2 cells with genetic deletion of *TRIB1* was considered as a possible factor to explain the lack of KO effect on TRIB1 inducer BRD0418 mediated suppression of lipogenic genes^18^. Nevertheless, this in vitro study in HepG2 cells failed to demonstrate a causal relationship between TRIBI induction and the suppression of lipogenic genes by BBR treatment.

In summary, here we have demonstrated that BBR-mediated hypolipidemic effects are mediated through LDLR-dependent and independent cellular mechanisms. Future studies using *Trib1* KO mice will be required to clearly validate the contribution of TRIB1 in BBR-mediated hypolipidemic effect. Since LDLR, PCSK9 and TRIB1 are all important regulators of circulating LDL-C and the CVD risk, their concurrent regulations by the natural lipid lowering compound BBR warrant further investigations.

## Materials and Methods

### Animals, diets and BBR treatment

All animals were maintained on a 12 h light-dark cycle and were allowed free access to food and water. Experimental protocols used in this study were all approved by the Institutional Animal Care and Use Committee of the VA Palo Alto Health Care System and all studies were conducted according to the guidelines of the Institutional Animal Care and Use Committee of the VA Palo Alto Health Care System. BBR (Sigma, St. Louis, USA) was suspended in 0.5% carboxyl-methyl cellulose (vehicle) at a concentration of 15 mg/ml and sonicated at 4 °C in a Bioruptor 300 instrument (Diagenode, Inc.). Adenoviral vectors (Ad-hTRIB1, Ad-m-shTRIB1, Ad-sh-hTRIB1 and Ad-GFP) were purchased from Vector Biolabs (Philadelphia, PA).

LDLR WT FVB mice (8–10 weeks old) were used in the study. For NCD experiments, mice were divided into two groups (n = 10, 5 M/5 F per group) and were administered with a daily dose of BBR (200 mg/kg) or vehicle by oral gavage for 14 days. For hyperlipidemic experiments, mice were fed a rodent HFHCD containing 1.25% cholesterol (# D12108C, Research Diet, Inc.) for two weeks. Mice were then divided into two groups (n = 10, 5 M/5 F per group) and were given a daily dose of BBR (200 mg/kg) or vehicle control by oral gavage for 14 days. Serum samples were collected after a 4 h fasting (9AM to 1PM) before and after the drug treatment.

In another study, LDLR deficient mice on the C57BL/6 J background (B6.129S7-Ldlr^tm1Her^/J, stock no. 002207, hence called *Ldlr*^−/−^) and 8–10 weeks of age were purchased from the Jackson Laboratory. The mice were fed a standard rodent chow diet containing 4.5% fat (No. 5001: Lab Diets, St. Louis, MO) ad libitum. For the experiment, six male *Ldlr*^−/−^ mice were randomly divided in two groups (n = 3 per group) and one group was administered with BBR (150 mg/kg) by oral gavage for 7 days. The control group received vehicle. After the last dosing, all animals were fasted 4 h (9AM to 1PM) before sacrificing for collection of serum and liver tissues. Sera and liver tissues were stored at −80 °C until analysis.

### Measurement of serum lipids

Fasting blood samples were collected from the retro-orbital plexus using heparinized capillary tubes under anesthesia (2–3% isoflurane and 1–2 L/min oxygen) and serum was separated by centrifugation at 8000 g for 10 min and stored at −80 °C. Serum levels of TG, TC and LDL-C were determined enzymatically using colorimetric assay kits purchased from Stanbio Laboratory (Texas, USA) with duplicate measurement for each sample.

### Measurements of hepatic lipids

Frozen liver samples were homogenized in 1 ml chloroform/methanol (2:1), followed by lipid extraction as described by Folch *et al*.^27^. Lipid extracts were dried and dissolved in isopropanol containing 10%Titon X-100 solution. Hepatic concentrations of cholesterol and triglycerides were measured using commercial kits (Stanbio Laboratory) and normalized per gram liver weight.

### Cell culture and treatment

Human hepatoma HepG2 cells were obtained from American Type Culture Collection (Manassas, VA, USA). Human primary hepatocytes (HPH) were obtained from Invitrogen; Thermo Fisher Scientific, Inc. (Waltham, MA, USA). Mouse primary hepatocytes (MPH) were isolated from male C57BL/6 J mouse at San Francisco General Hospital Liver Center. HepG2 cells were cultured in Minimum Essential Medium supplemented with 10% fetal bovine serum (FBS) and 1% Penicillin/Streptomycin solution at 37 °C under a 5% CO2 atmosphere. For the experiments, HepG2 cells were cultured in 12-well plate overnight in MEM containing 0.5% FBS and were treated with BBR (40 μM) at different time intervals prior to cell lysis for protein and RNA extraction. HPHs were seeded on collagen coated plates at a density of 1 × 10^5^ cells/well in 24-well plates in Williams E Medium supplemented with a Cell Maintenance Cocktail (Cell Maintenance Supplement Pack, Invitrogen). After overnight seeding, cells were treated with BBR for 3 h before total RNA isolation.

### Adenoviral transduction

MPH or HepG2 cells were seeded at 1 × 10^5^ cells/well in 24-well plates overnight. The next day, cells were transduced with the adenovirus at various multiplicity of infection (MOI) in 0.5 ml medium containing 0.5% FBS for 8 h. Then viral-containing medium was replaced by fresh complete medium and cells were cultured for 72 h before isolation of total RNA or total lysate.

### Constructions of human *TRIB1* promoter luciferase reporters

For generation of *TRIB1* promoter reporter, a DNA fragment of 967 bp covering human *TRIB1* proximal promoter region from −840 to +127 relative to the 5’end of exon 1 was amplified from HepG2 genomic DNA by using the forward primer 5′-GAGGCTGGGGAGGGAGTAGG-3′ and Reverse primer 5′-CCAAAGCGATGAGTCTCCAGC-3′. The amplified fragment was cloned into Topo 2.1 vector, followed by subcloning into pGL3-basic at the Kpn1 and Xho1 sites to yield the promoter reporter p-hTRIB1-Luc. After transformation and propagation in *E. coli*, six independent clones were sequenced to verify the sequence and orientation of the promoter fragment.

### Transient transfection and luciferase activity

HepG2 cells were seeded in 96-well culture plate (3 × 10^4^cells/well) and transfected with 100 ng of p-hTRIB1-Luc plasmid or pGL3-basic vector, and co-transfected with 10 ng of pCMV-β-Gal as an internal control by using Polyjet transfection reagent (SignaGen Laboratories, Gaithersburg, MD). One day post-transfection, cells were starved overnight in 0.5% FBS containing MEM and subsequently treated with BBR at a dose of 40 μM for 8 h or 24 h in the absence or presence of MEK1 inhibitor U0126. At the end of treatment time course, cells were lysed with 100 μl reporter lysis buffer with 50 μl cell lysate for measuring β-galactosidase activity by using β-Galactosidase Enzyme Assay System (Promega) and the remaining 50 μl of lysate for firefly luciferase activity assay (Luciferase Assay System (Promega). Absolute luciferase activity was divided by β-galactosidase activity to correct for transfection efficiency. Triplicate wells were assayed for each transfection condition.

### Western blotting and antibodies

Total lysates of cells and tissues were obtained by lysis in RIPA buffer with a protease inhibitor cocktail and a phosphatase inhibitor cocktail (Roche Diagnostics Corporation, USA). For Western blotting, liver homogenates (50–70 μg/well) or HepG2 lysates (20 μg/well) were separated by SDS polyacrylamide gel electrophoresis and transferred to a nitrocellulose membrane (Bio-Rad, USA), which was probed with the primary antibodies overnight at 4 °C. After 1 h incubation with secondary antibodies, proteins were visualized by using SuperSignal West Femto Chemiluminescent Substrate (Thermo Scientific) and a FluorChem E imaging system (ProteinSimple, USA). Supplemental Table 1 lists the primary and secondary antibodies used in this study.

### Total RNA isolation and quantitative real-time PCR

Total RNA was isolated from liver tissues and hepatic cells using RNeasy plus mini kit (Qiagen) according to manufacturer’s instructions. cDNA was synthesized from total RNA using the High Capacity cDNA Reverse Transcription Kits and real-time PCR were conducted by using SYBR green qPCR master mix (Applied Biosystems). PCR amplification was performed in triplicates in a 384-well plate for each cDNA sample on an ABI PRISM 7000 sequence-detection system (Applied Biosystems). Target mRNA expression in each sample was normalized to the house keeping gene *GAPDH*. The 2^−ΔΔCt^ method was used to calculate relative mRNA expression levels. Primer sequences of mouse and human genes used in real-time PCR are listed in Supplemental Table 2.

### siRNA transfection

On-TARGETplus Human *TRIB1* siRNA-SMARTpool (Cat. # L-003633-00-0005) and control siRNA ON-TARGETplus Non-targeting pool (Cat.# D-001810-10-05) were purchased from Dharmacon. HepG2 cells were seeded overnight in 12 well plates and siRNAs were transfected into HepG2 cells using transfection reagent (Dharmacon) at a final concentration of 30 nM. After 8 h transfection, medium was replaced with fresh MEM medium containing 10% FBS for 48 h, cells were treated with either vehicle control (DMSO) or BBR (40 μM) for the last 12 h prior to cell lysis. siRNA sequences are listed in Supplemental Table 2.

### Statistical analyses

Data are presented as the Mean ± SEM. Results were analyzed by Student’s t-test (two-tailed) or ANOVA with Tukey’s multiple comparisons post-test, when multiple comparisons to control group were made. Statistical significance was defined as p < 0.05.

## Supplementary information

supplemental materials

## Data Availability

All data generated or analyzed during this study are included in this published article (and its Supplementary Information files).
