# Supplementary material for: Berberine decreases plasma triglyceride levels and upregulates hepatic TRIB1 in LDLR wild type mice and in LDLR deficient mice
Source: Sci Rep. 2019 Oct 30;9:15641. doi: 10.1038/s41598-019-52253-y (PMC6821852; doi:10.1038/s41598-019-52253-y)
Supplement: Supplementary file 1 — supplemental materials [file 41598_2019_52253_MOESM1_ESM.docx]

**Berberine decreases plasma triglyceride levels and upregulates hepatic TRIB1 in LDLR wild type mice and in LDLR deficient mice**

**Amar Bahadur Singh and Jingwen Liu***

**Department of Veterans Affairs Palo Alto Health Care System, Palo Alto, California 94304**

**Supplemental tables and figures**

**Supplemental Table 1. List of antibodies**

| **Antibody** | **Species** | **Catalog No.** | **Vendor** |
| --- | --- | --- | --- |
| LDLR | Rabbit | 3839-100 | Bio-vision |
| TRIB1 | Rabbit | Ab137717 | Abcam |
| FAS | Mouse | Sc-55580 | Santa Cruz |
| SCD1 | Mouse | Sc-14720 | Santa Cruz |
| P-ERK | Rabbit | #4370S | Cell Signaling |
| ERK | Rabbit | #4695S | Cell Signaling |
| DGAT1 | Rabbit | Ab54037 | Abcam |
| C/EBPα | Rabbit | Sc-61 | Santa Cruz |
| CPT1α | Mouse | Ab128568 | Abcam |
| β-Actin | Mouse | A1978 | Sigma |
| Anti-Fibronectin | Mouse | F3648 | Sigma |
| Anti-rabbit HRP-conjugate | Rabbit | 7074P2 | Cell Signaling |
| Anti-mouse HRP-conjugate | Mouse | 7076P2 | Cell Signaling |

**Supplemental Table 2.** Real-time PCR primer sequences

| **Gene names** | **Forward** | **Reverse** |
| --- | --- | --- |
| **Human** | | |
| LDLR | GACGTGGCGTGAACATCTG | CTGGCAGGCAATGCTTTGG |
| PCSK9 | AGGGGAGGACATCATTGGTG | CAGGTTGGGGGTCAGTACC |
| TRB1 | CTTAGGAAGTTCGTCTTCTCCAC | GGCAGCCATGTTTGTCTGAC |
| TRB2 | CTTTTGCCTGTCTGCTCATAGT | ATAGCTTCGCTCAAAGAACACA |
| TRB3 | TGCGTGATCTCAAGCTGTGT | GCTTGTCCCACAGGGAATCA |
| ACC1 | GAGGGCTAGGTCTTTCTGGAAG | CCACAGTGAAATCTCGTTGAGA |
| SCD1 | CCCGACGTGGCTTTTTCTTC | GCCAGGTTTGTAGTACCTCCTC |
| DGAT1 | CCCCCAACAAGGACGGAGAC | GGCATTGCTCAAGATCAGCATC |
| DGAT2 | GTTTCGCCCCATGCATCTTC | ACAGGTCGATGTCTTGCTGG |
| C/EBPα | TTGTATCTGGCCTCTGTGCC | GCCGACGGAGAGTCTCATTT |
| CPT1α | TGAGCGACTGGTGGGAGGAG | GAGCCAGACCTTGAAGTAGCG |
| GAPDH | ATGGGGAAGGTGAAGGTCG | GGGGTCATTGATGGCAACAATA |
| **Mouse** | | |
| TRIB1 | CCCGAGATCCTCAACACTACT | CATGAAAGGGGTATCGTCCGA |
| TRIB2 | ATACACAGGTCTACCCCTATCAC | ATGCGACAAGTTCGGAGTCTC |
| TRIB3 | CAGGAAGAAACCGTTGGAGTT | CCAAAAGGATATAAGGCCCCAGT |
| ACC1 | AGCACAGCTCCAGATTGCCA | GGAGATACCCCATACATCATAC |
| SCD1 | CTGCAGGTTGTGCTAGATGGGATGG | GCCTGGGGTCTTTGGTAAGTAGGC |
| FASN | GTGATAGCCGGTATGTCGGG | TAGAGCCCAGCCTTCCATCT |
| LDLR | ACCTGCCGACCTGATGAATTC | GCAGTCATGTTCACGGTCACA |
| PPARγ | TGTGAGACCAACAGCCTGAC | CCGCTTCTTTCAAATCTTGTCTGT |
| SREBP1c | CAAGGCCATCGACTACATCCG | CACCACTTCGGGTTTCATGC |
| CPT1α | GGCCATCTGTGGGAGTATGT | ACTGTAGCCTGGTGGGTTTG |
| ACOX1 | TCGGCAAAAACTTCCAAATC | GGCTGTGTATCACAAACTCCTG |
| DGAT1 | TGGTAGTGGGCCCAAGGTAG | GAATCTTGCAGACGATGGCAC |
| DGAT2 | GGCTACGTTGGCTGGTAACT | TCTTCAGGGTGACTGCGTTC |
| GPAT1 | CAACACCATCCCCGACATC | ACTGTTCGCCATTGTAGTGACCTT |
| C/EBPα | GAGGGGAGGGACTTAGGTGT | GGAGGTGCAAAAAGCAAGGG |
| MLXIPL | TGGGTGTTCAGCATCCTCATC | CAGCCAGGCCAGTGAGGTCT |
| TNFα | ACTGAACTTCGGGGTGATCG | CTTGGTGGTTTGCTACGACG |
| ApoB | TTGGCAAACTGCATAGCATCC | TCAAATTGGGACTCTCCTTTAGC |
| IL6 | CACTTCACAAGTCGGAGGCT | CTGCAAGTGCATCATCGTTGT |
| MTTP | CCAGGGCTTTTGCCTTGAAC | GAGGACCTGTCCCACAATGG |
| IL1β | TGCCACCTTTTGACAGTGATG | TGATGTGCTGCTGCGAGATT |
| GAPDH | ATGGTGAAGGTCGGTGTGAA | ACTGGAACATGTAGACCATGTAGT |
| **Cloning Primers** | | |
| Hu-TRIB1Promoter | GAGGCTGGGGAGGGAGTAGG | CCAAAGCGATGAGTCTCCAGC |
| **ON-TARGETplus Human TRIB1 (10221) siRNA-SMART pool (Cat. # L-003633-00-0005)** | | |
| J-003633-07 | GCAAGGUGUUUCCCAUUAA | |
| J-003633-08 | CUAGAAGACACACACAUAA | |
| J-003633-09 | CGGAAAGGCUGCGGACGUU | |
| J-003633-10 | GAACCCAGCUUAGACUAGA | |
| **ON-TARGETplus Non-targeting pool sequences (Cat. # D-001810-10-05)** | | |
|  | UGGUUUACAUGUCGACUAA | |
|  | UGGUUUACAUGUUGUGUGA | |
|  | UGGUUUACAUGUUUUCUGA | |
|  | UGGUUUACAUGUUUUCCUA | |

**Supplemental Figure 1 (A-C)**


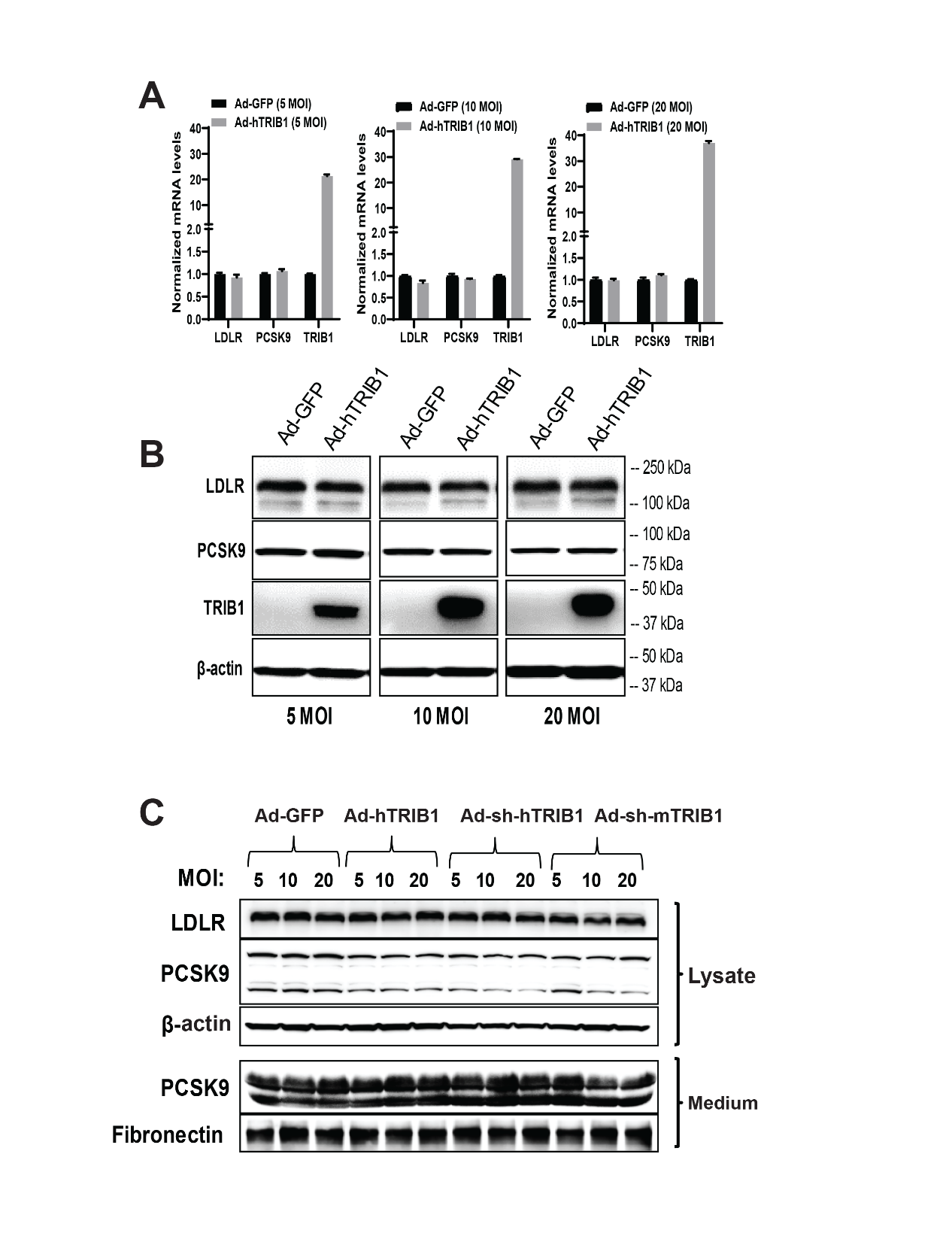


**Supplemental Figure 1D**

**Supplemental Figure 1.** **Lack of effects of TRIB1 overexpression or knockdown on LDLR and PCSK9 protein levels.** MPHs were transduced with control adenovirus Ad-GFP or Ad-hTRIB1 expressing human TRIB1 in different MOIs for 3 days and total RNA and protein lysates were isolated.

**(A)** Mouse *Ldlr* and *Pcsk9* and human *TRIB1* mRNA levels were determined by qPCR.

**(B)** Cellular protein levels of mouse LDLR, PCSK9 and human TRIB1 were assayed by Western blotting.

**(C)** HepG2 cells were transduced with different MOIs of control adenovirus (Ad-GFP), Ad-hTRIB1, Ad-sh-hTRIB1 expressing a shRNA targeting human *TRIB1* mRNA and Ad-sh-mTRIB1 targeting mouse *Trib1* mRNA as another control. After 3 days, medium and total cell lysates were collected for Western blot analysis.

**(D)** Signals in Western blot analysis was quantified to determine LDLR and intracellular PCSK9 levels with β-actin as the normalizing control. PCSK9 abundance in medium was normalized with fibronectin.

**Supplemental Figure 2.**

**Supplemental Figure 2.** HepG2 cells were transduced with different MOIs of control adenovirus (Ad-GFP) or Ad-hTRIB1 in (A). and with control virus Ad-shLacZ or Ad-sh-hTRIB1 with different MOIs. After 3 days, total RNA was isolated and analyzed for LDLR, TRIB1 and GAPDH mRNA expression levels by qPCR.
